# Super sub-wavelength patterns in photon coincidence detection

**DOI:** 10.1038/srep04068

**Published:** 2014-02-17

**Authors:** Ruifeng Liu, Pei Zhang, Yu Zhou, Hong Gao, Fuli Li

**Affiliations:** 1 MOE Key Laboratory for Nonequilibrium Synthesis and Modulation of Condensed Matter, and Department of Applied Physics, Xi'an Jiaotong University, Xi'an 710049, People's Republic of China

## Abstract

High-precision measurements implemented with light are desired in all fields of science. However, light acts as a wave, and the Rayleigh criterion in classical optics yields a diffraction limit that prevents obtaining a resolution smaller than the wavelength. Sub-wavelength interference has potential application in lithography because it beats the classical Rayleigh resolution limit. Here, we carefully study second-order correlation theory to establish the physics behind sub-wavelength interference in photon coincidence detection. A Young's double slit experiment with pseudo-thermal light is performed to test the second-order correlation pattern. The results show that when two point detectors are scanned in different ways, super sub-wavelength interference patterns can be obtained. We then provide a theoretical explanation for this surprising result, and demonstrate that this explanation is also suitable for the results found for entangled light. Furthermore, we discuss the limitations of these types of super sub-wavelength interference patterns in quantum lithography.

Quantum lithography was first proposed by Boto *et al.*^1^ and allows an N-times smaller spacing of interference fringes than the classical Rayleigh limit^2^ for spatial resolution. To date, true laboratory verification of quantum lithography remains extremely challenging because no lithographic materials exist that are capable of N-photon absorption lithographic materials^3^. The alternative experiment to demonstrate quantum lithography uses N detectors operating in coincidence to mimic the effect of a true N-photon absorbing resist. In interferometric lithography, coherent light beams shine on a mask and form an interference pattern. If the spacing of the interference fringes is smaller than the wavelength *λ*, which is the classical Rayleigh limit^2^ (the Rayleigh limitation is smaller than *λ*, here we use *λ* for easy comparison to sub-wavelength), one obtains the sub-wavelength interference pattern, which can be used in lithography. This sub-wavelength interference pattern has been obtained using entangled light^4^ by simultaneously scanning two point detectors in the same directions and also using thermal light^5^,^6^,^7^ by simultaneously scanning the two point detectors in opposite directions. However, it is unclear why the sub-wavelength interference pattern can be obtained only when the two point detectors are scanned in those ways, and it is unknown whether other, better scanning approaches exist that provide a spatial resolution smaller than *λ*/2. In our study, we find that using the appropriate scanning method, even super sub-wavelength interference patterns can be achieved. This surprising result encourages us to consider the physics behind this phenomenon and to determine whether we can achieve an interference pattern with arbitrarily high resolution. We address these questions in this manuscript and suggest how to obtain a full understanding of the second-order intensity correlation.

In interference and diffraction experiments with light, the observed quantity is the first-order correlation function of the light on a detection plane in the far-field region, 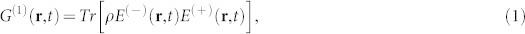
where *E*^(−)^(**r**, *t*) and *E*^(+)^(**r**, *t*) are operators for the negative and positive frequencies of the light field, *ρ* is the density matrix operator for the light source, and **r** is a transverse coordinate vector on the detection plane. The measured first-order correlation in Eq. (1) directly represents the distribution of light intensity on the detection plane.

In addition to the first-order correlation, light can have a second-order correlation, which is defined as
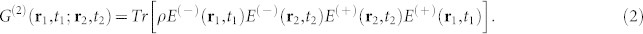
In 1956, Hanbury-Brown and Twiss (HBT)^8^,^9^ first observed the second-order intensity correlation of light in astronomy. This investigation arouse research interest into the second-order correlation properties of light^10^,^11^, and inspired Glauber's work on quantum optics^12^,^13^. The HBT experiment can be physically explained by either the classical statistical correlation of the intensity fluctuations^10^ or by the interference of two-photon (multiphoton) probability amplitude^14^. Although our understanding of the physical mechanism behind the HBT experiment has long been debated, the second-order correlation of light has been widely applied in various fields, such as nonlocal imaging and interference^15^,^16^,^17^,^18^,^19^,^20^,^21^, sub-wavelength interference^4^,^5^,^6^,^7^, and quantum lithography^3^,^22^,^23^,^24^.

## Results

**Figure 1:**
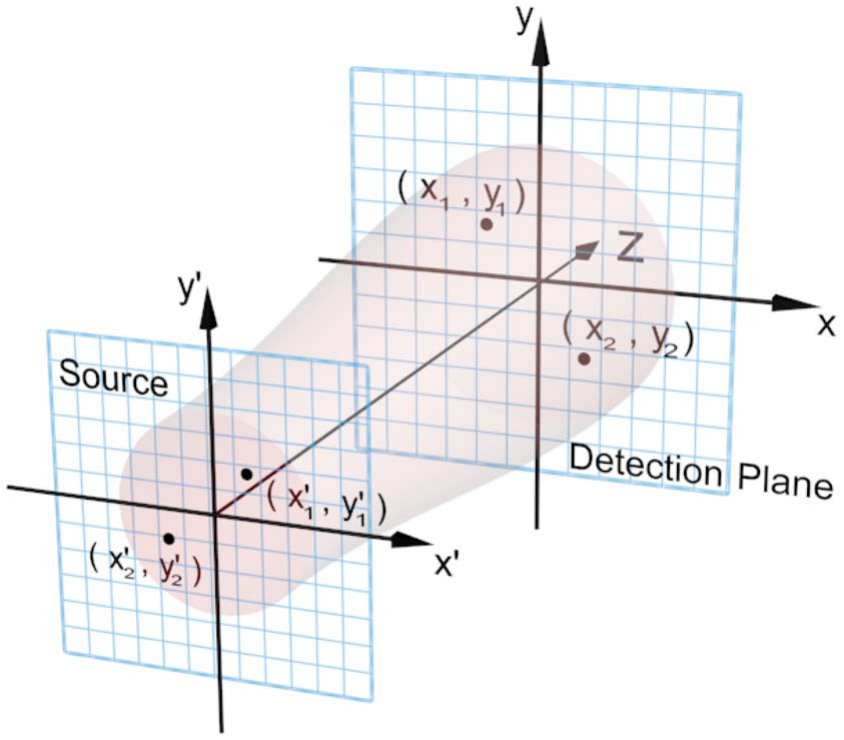
Schematic of the correspondence between the source and the detection plane.

In Fig. 1, we show the point-to-point correspondence between the source and the detection plane; this correspondence can be described by the impulse response function *h*(**r**, **r**′). If the source is thermal light, the second-order correlation function on the detection plane can be written as^16^
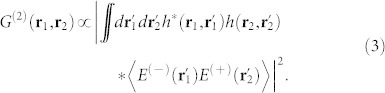


Under the paraxial approximation and the assumption that the source is covered by an object with a transmission function *T*(**r**′), the impulse response function can be expressed as
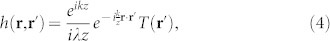
where *k* = 2*π*/*λ* is the wave number. If the light source is fully incoherent, as it is for thermal light, the first-order correlation function of light just emitted from the source 
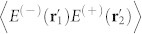
 can take the form 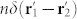, where *δ*(**r**) is the Dirac delta function, and *n* is the mean photon number. Here, we have assumed that the intensity distribution of the incoherent light on the source plane is uniform^25^. Then, the second-order correlation function in Eq. (3) can be rewritten in the form
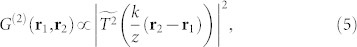
where 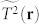 is the Fourier transformation of *T*^2^(**r**). This result shows that a well-defined Fourier-transform image of the transmittance of an object can be extracted from the second-order correlation function.

Eq. (5) clearly shows that the second-order correlation function is a four-variable function of the transverse coordinates (*x*_1_, *y*_1_) and (*x*_2_, *y*_2_) of the detection plane. Simply but without loss of generality, a one-dimensional object such as a double-slit is usually employed to force the correlation function to become a two-variable function. For a one-dimensional symmetric double-slit placed along the y-axis, the correlation function is independent of the coordinates *y*_1_ and *y*_2_. In this way, the second-order correlation function in Eq. (5) can be further simplified to the form
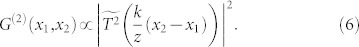


## Experimental test with pseudo-thermal light

**Figure 2:**
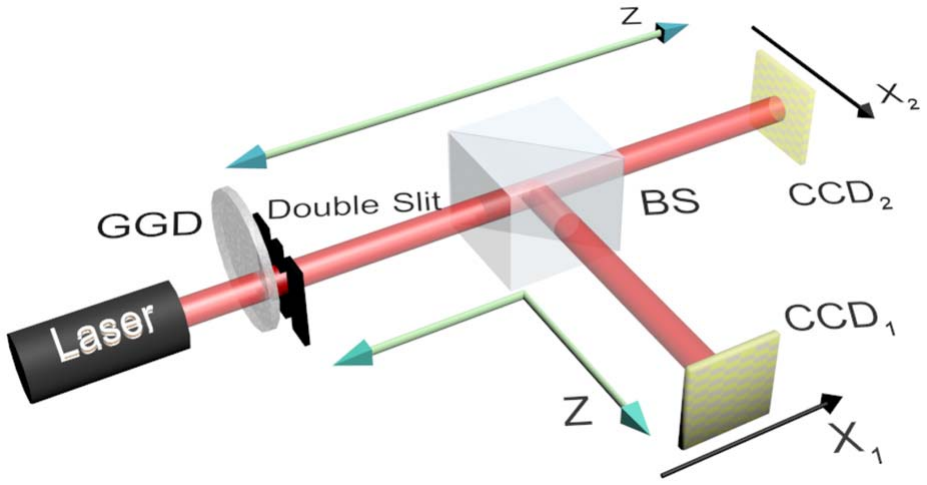
Schematic for the second-order correlation measurement of a double-slit with charge-coupled devices (CCDs). The rotating ground glass disk (GGD) and the double-slit are placed as close together as possible. The two CCDs are placed in the Fraunhofer diffraction region with respect to the double-slit. The CCD is a 1, 040 × 1, 392 array of 4.65 × 4.65 *µ*m^2^ pixels, and the measurement is made with an exposure time of 0.2 ms.

The experimental setup is shown in Fig. 2. Pseudo-thermal light is produced by a semiconductor laser with a wavelength *λ* = 457 nm and a rotating ground glass disk (GGD). The angular velocity of the GGD is maintained at *ω* = *π* rad/s. A double-slit is placed immediately after the GGD. The width of each slit is *a* = 0.038 mm, and the distance between centres of the two slits is *d* = 0.12 mm. The diffracted light is split into two parts after the double-slit using a beam splitter (BS) and is then recorded by two charge-coupled devices (CCDs). Both of the CCDs are located at *z* = 23 cm behind the double-slit; therefore the CCDs are placed in the Fraunhofer diffraction region with respect to the double-slit.

**Figure 3:**
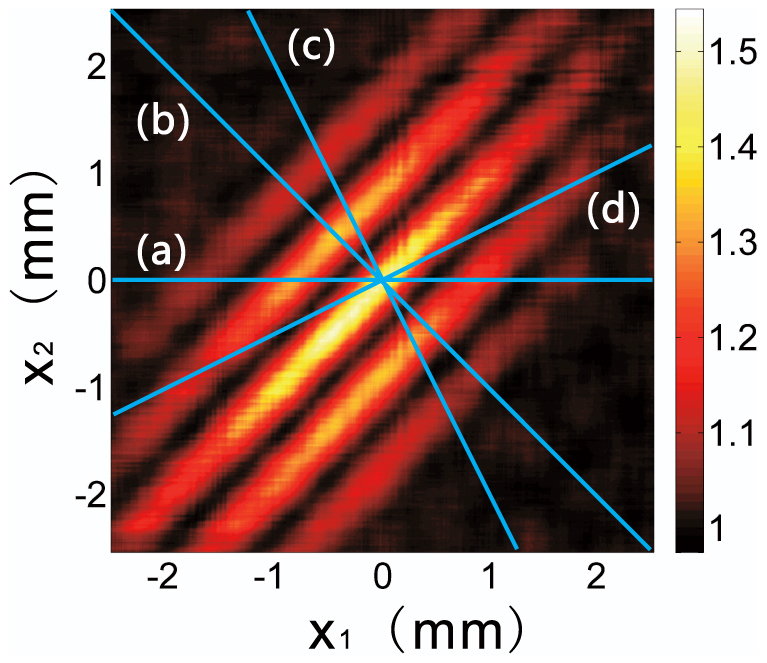
Second-order correlation function Eq. (6) recorded using two CCDs. *x*_1_ and *x*_2_ are the horizontal ordinates on CCD_1_ and CCD_2_, respectively. The lines represent various scanning methods for the detectors: line (a) for *x*_1_ = *x* and *x*_2_ = 0; line (b) for *x*_1_ = *x* and *x*_2_ = −*x*; line (c) for *x*_1_ = *x* and *x*_2_ = −2*x*; line (d) for *x*_1_ = *x* and *x*_2_ = *x*/2.

As shown above, the second-order correlation function Eq. (6) describes the correlation between two arbitrary points on CCD_1_ and CCD_2_. Here, we extract the relevant row data from the two CCDs, and construct the second-order correlation function. The result is shown in Fig. 3, where *x*_1_ and *x*_2_ are the horizontal coordinates on CCD_1_ and CCD_2_, respectively. Here, we use colour to represent the value of the second-order correlation function. This second-order correlation function is the two-dimensional second-order interference pattern of the double-slit.

**Figure 4:**
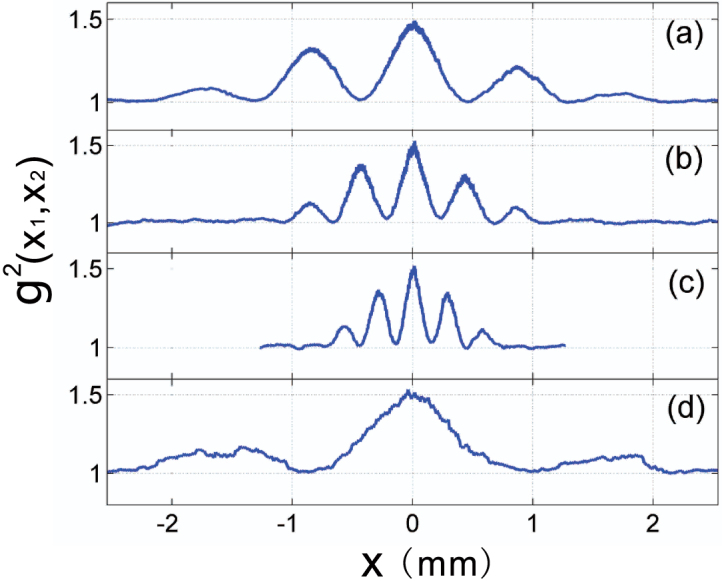
Cross-sectional curves of (a), (b), (c), and (d) represent the corresponding lines in Fig. 3.

A cross-sectional curve with arbitrary spatial resolution can be obtained by choosing the correct line on the two-dimensional figure. Line (a) in Fig. 3 describes the case when one point detector on the CCD_2_ plane is fixed and another point detector on the CCD_1_ plane is moved along the *x*_1_ direction. The curve obtained along this line, is identical to the traditional Young's interference pattern, as shown in Fig. 4(a). The distance from the zeroth-order peak to the first-order peak in Fig. 4(a) is 0.89 mm, consistent with the theoretical value of 0.88 mm. Synchronous scanning with two point detectors in opposite directions corresponds to line (b) in Fig. 3 and yields an interference pattern with peak spaces of 0.44 mm, as shown in Fig. 4(b). This result is the sub-wavelength interference pattern described in Ref. 5, 6.

Obviously, the scanning method is not limited on the above two cases. We can choose any scanning method that corresponds to a line in Fig. 3 to obtain a diffraction pattern with arbitrary-wavelength resolution. For example, along line (c) in Fig. 3, representing a scanning method with *x*_1_ = *x*, and *x*_2_ = −2*x*, we obtain a narrower interference pattern than the sub-wavelength pattern, as shown in Fig. 4(c). The peak spacing in Fig. 4(c) is 0.29 mm, which corresponds to a spatial resolution of *λ*/3. If we set *x*_1_ = *x*, and *x*_2_ = *x*/2, as represented by line (d) in Fig. 3, the resulting data displays a spatial resolution of 2*λ* (Fig. 4(d)). The experimental results obtained here fit the theoretical prediction obtained using Eq. (6) well. From the above discussion, we conclude that one-dimensional cross-sectional curves of the second-order correlation function can have super sub-wavelength behaviour if the appropriate detector scanning method is chosen.

## Results for an entangled light source

The above conclusion can be applied to sub-wavelength interference from an entangled light source. In this case, Angelo *et al.*^4^ showed that an interference pattern with enhanced spatial resolution could be constructed, as predicted by Boto *et al.*^1^. In this experiment, a double-slit is placed immediately after the nonlinear crystal to ensure that two photons are generated simultaneously at the upper or the lower-slit. Two point detectors move in the same direction synchronously and operate in coincidence mode to mimic the effect of a true two-photon absorbing resist. Angelo *et al.* found that the two-photon double-slit interference pattern is 2-fold narrower than the traditional pattern obtained using coherent light. If one assumes that the entangled photon pairs are generated simultaneously at either the upper or lower-slit and propagate to the detection plane independently, the coincidence count at the detection plane can be approximately expressed in the form^26^,^27^,^28^
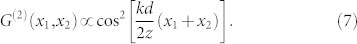


**Figure 5:**
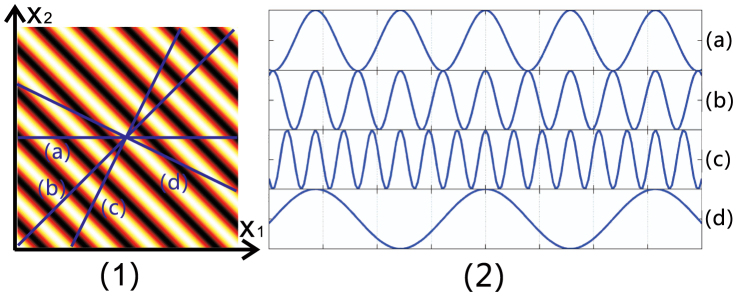
Second-order interference pattern for entangled light. (1) Two dimensional pattern of second-order correlation function Eq. (7). (2) (a), (b), (c), and (d) represent the cross-sectional curves that are correspond to the labelled lines in (1).

For simplicity, the single-slit diffraction function has been ignored. As shown in Eq. (7), the second-order correlation function is a two-dimensional function of the detection plane coordinates. In Fig. 5(1), the correlation function in Eq. (7) is plotted. This pattern is similar to the experimental result obtained by Peeters *et al.*^29^.

As in the analysis for thermal light, line (a) in Fig. 5(1) represents an interference pattern that has the same spatial resolution as the normal Young's double-slit experiment. The sub-wavelength result obtained by D'Angelo^4^ can be rebuilt along line (b) in Fig. 5(1). The cross-sectional curves along these two lines are plotted in Fig. 5(2) (a)-(b). Lines (c) and (d) represent detector scanning methods with (*x*_1_ = *x*, *x*_2_ = 2*x*) and (*x*_1_ = *x*, *x*_2_ = −*x*/2), respectively. In Fig. 5(2) (c)-(d), the cross-sectional curves corresponding to lines (c) and (d) are plotted with peak spacings of *λ*/3 and 3*λ*, respectively. As described in Ref. 28, the two photons that are simultaneously diffracted by a double-slit will propagate independently; thus, the photons in coincidence measurement are not limited to one line only but completely fill the entire detection plane. Therefore, the one-dimensional cross-sectional curve deduced from the two-dimensional second-order correlation function may also have arbitrarily narrow peak distances when entangled light is used as the source.

## Discussion

Based on the above results for both thermal and entangled light, it is clear that a cross-sectional curve can be constructed with arbitrary spatial resolution by choosing the scanning method for the detectors in the detection plane. However, cross-sectional curves represent only part of the information obtained from the second-order interference. To obtain all of the information, we should consider all possible combinations of arguments for the second-order correlation function. For example, when including all possible variations of the two arguments, the full picture of the second-order interference of a double-slit is a two-dimensional pattern.

It remains challenging to achieve a true laboratory demonstration of quantum lithography due to the absence of lithographic (*N*-photon absorption) materials, although proof-of-principle experiments that display certain aspects of quantum lithography have been performed. In these proof-of-principle experiments, *N* photons should appear at same point on the *N* detectors simultaneously because the *N*-photon absorber is simulated by *N*-fold coincidence. From this perspective, we can see that if *N* detectors scan with different paces and in different directions, it is impossible to take advantage of the resulting super sub-wavelength patterns to achieve quantum lithography. In other words, for thermal light, the second-order interference pattern becomes flat when two detectors scan in the same directions (*x*_1_ = *x*_2_), and this method clearly can-not be used for writing sub-Rayleigh structures. However, for entangled light, a sub-wavelength interference pattern is obtained when two detectors are scanned with *x*_1_ = *x*_2_ (as shown in Fig. 5(2) (b)). Thus, an entangled light source has potential for application in quantum lithography, although the efficiency obtained may be an obstacle^27^,^28^,^30^. We also note that measurement of the two-dimensional pattern is not limited in the space-momentum system. For example, this approach can be applied to entropic entanglement measurements of a photon's orbital angular momentum^31^.

In summary, based on the well-known fact that the second-order interference is in general a high-dimensional pattern instead of a one-dimensional curve, we showed that in double-slit second-order interference experiments with either thermal or entangled light, when two point detectors move in a certain way on the signal and reference detection planes, the result obtained from the coincidence count of the detectors is merely a cross-sectional curve of the two-dimensional interference pattern. In principle, one can construct a cross-sectional curve of arbitrary spatial resolution by appropriately choosing the scanning method for the two point detectors on the detection plane. Furthermore, we find that it is impossible to apply this type of super sub-wavelength interference to quantum lithography using thermal light. Our theory and experimental method for second-order interference may provide a deep understanding of higher-order correlations and holds promise for the development of applications of these correlation.

## Methods

The two variables *x*_1_ and *x*_2_ described in Eq. (6), represent the positions of two detectors. Thus, all the information available from the second-order correlation function can be obtained only by considering all possible combinations of *x*_1_ and *x*_2_ in the detection plane. In previous sub-wavelength interference experiments using a thermal light source^5^,^6^, *λ*/2 spatial resolution cross-sectional curves were obtained when two point detectors were scanned in opposite directions (*x*_1_ = *x*, *x*_2_ = −*x*). However, there are no mathematical or physical reasons to limit the scanning methods for two detectors to the coordinates *x*_1_ and *x*_2_ in Eq. (6). For example, if the two point detectors move in the detection plane along *x*_1_ = *x*, *x*_2_ = *x*/2, one can obtain the cross-sectional curve with spatial resolution 2*λ*. In contrast, if the two point detectors move in the detection plane along *x*_1_ = *x*, *x*_2_ = −2*x*, one can obtain cross-sectional curve with spatial resolution *λ*/3. In general, the cross-sectional curve with spatial resolution *Nλ* or 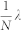 (where *N* > 0) can be obtained when the two point detectors scan in the detection plane along *x*_1_ = *x*, 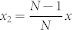 or *x*_1_ = *x*, *x*_2_ = (1 − *N*)*x*, respectively. Therefore a cross-sectional curve with arbitrary spatial resolution can be obtained merely by choosing an appropriate scanning method. The result that the physical quantity *G*^(2)^ is not independent of the scanning method appears surprising. However, noting the second-order correlation function Eq. (6), we can find the reason for this effect—the second-order correlation pattern is a two-dimensional surface rather than a one-dimensional curve. To further clarify our argument, we perform a second-order interference experiment using pseudo-thermal light with a setup similar to that employed in Ref. 5, 6 as described in the Results section.

## Author Contributions

R.L. constructed and operated the experiment, and collected the data. P.Z. and F.L. devised and designed the experiment. Y.Z. and H.G. performed the theoretical calculations. All authors contributed to the manuscript.

## References

[b1] Boto A. N. *et al.* Quantum interferometric optical lithography: exploiting entanglement to beat the diffraction limit. Phys. Rev. Lett. 85, 2733–2736 (2000). 10991220 10.1103/PhysRevLett.85.2733

[b2] Rayleigh L. Investigations in optics, with special reference to the spectroscope. Philos. Mag. 8, 261 (1879).

[b3] Boyd R. W. & Dowling J. P. Quantum lithography: status of the field. Quantum Inf. Process 11, 891–901 (2012).

[b4] D'Angelo M., Chekhova M. V. & Shih Y. Two-photon diffraction and quantum lithography. Phys. Rev. Lett. 87, 013602 (2001). 11461466 10.1103/PhysRevLett.87.013602

[b5] Scarcelli G., Valencia A. & Shih Y. Two-photon interference with thermal light. Europhys. Lett. 68, 618–624 (2004).

[b6] Xiong J. *et al.* Experimental observation of classical sub-wavelength interference with thermal-like Light. Phys. Rev. Lett. 94, 173601 (2005). 15904288 10.1103/PhysRevLett.94.173601

[b7] Zhai Y. H., Chen X. H., Zhang D. & Wu L. A. Two-photon interference with true thermal light. Phys. Rev. A 72, 043805 (2005).

[b8] Brown R. H. & Twiss R. Q. Correlation between photons in two coherent beams of light. Nature 177, 27–29 (1956).

[b9] Brown R. H. & Twiss R. Q. A test of a new type of stellar interferometer on Sirius. Nature 178, 1046–1048 (1956).

[b10] Brown R. H. Intensity Interferometer (Taylor Francis, London, 1974).

[b11] Mandel L. & Wolf E. Photon Correlations Optical Coherence and Quantum Optics (Cambridge University Press, Cambridge, 1995).

[b12] Glauber R. J. Photon correlations. Phys. Rev. Lett. 10, 84–86 (1963).

[b13] Glauber R. J. Coherent and Incoherent States of the Radiation Field. Phys. Rev. 131, 2766–2788 (1963).

[b14] Scarcelli G., Berardi V. & Shih Y. Can two-photon correlation of chaotic light be considered as correlation of intensity fluctuations? Phys. Rev. Lett. 96, 063602 (2006). 16605993 10.1103/PhysRevLett.96.063602

[b15] Pittman T. B., Shih Y. H., Strekalov D. V. & Sergienko A. V. Optical imaging by means of two-photon quantum entanglement. Phys. Rev. A 52, R3429–R3432 (1995). 9912767 10.1103/physreva.52.r3429

[b16] Cheng J. & Han S. Incoherent coincidence imaging and its applicability in X-ray diffraction. Phys. Rev. Lett. 92, 093903 (2004). 15089466 10.1103/PhysRevLett.92.093903

[b17] Gatti A., Brambilla E., Bache M. & Lugiato L. A. Ghost imaging with thermal light: comparing entanglement and classical correlation. Phys. Rev. Lett. 93, 093602 (2004). 15447100 10.1103/PhysRevLett.93.093602

[b18] Boyd R. W. & Dowling J. P. Special Issue: Quantum Imaging. Quantum Inf. Process 11, 887–1011 (2012).

[b19] Ribeiro P. S., Pádua S., da Silva J. M. & Barbosa G. A. Controlling the degree of visibility of Young's fringes with photon coincidence measurements. Phys. Rev. A 49, 4176–4179 (1994). 9910717 10.1103/physreva.49.4176

[b20] Fonseca E. J. S., Ribeiro P. S., Pádua S. & Monken C. H. Quantum interference by a nonlocal double slit. Phys. Rev. A 60, 1530–1533 (1999).

[b21] Fonseca E. J. S., Monken C. H. & Pádua S. Measurement of the de Broglie wavelength of a multiphoton wave packet. Phys. Rev. Lett. 82, 2868–2871 (1999).

[b22] Sciarrino F. *et al.* Experimental sub-Rayleigh resolution by an unseeded high-gain optical parametric amplifier for quantum lithography. Phys. Rev. A 77, 012324 (2008).

[b23] Tsang M. Quantum Imaging beyond the Diffraction Limit by Optical Centroid Measurements. Phys. Rev. Lett. 102, 253601 (2009). 19659073 10.1103/PhysRevLett.102.253601

[b24] Guerrieri F. *et al.* Sub-Rayleigh imaging via N-photon detection. Phys. Rev. Lett. 105, 163602 (2010). 21230971 10.1103/PhysRevLett.105.163602

[b25] Scarcelli G., Valencia A. & Shih Y. Experimental study of the momentum correlation of a pseudothermal field in the photon-counting regime. Phys. Rev. A 70, 051802 (2004).

[b26] Steuernagel O. On the concentration behaviour of entangled photons. J. Opt. B: Quantum Semiclass. Opt. 6, S606–S609 (2004).

[b27] Tsang M. Relationship between resolution enhancement and multiphoton absorption rate in quantum lithography. Phys. Rev. A 75, 043813 (2007).

[b28] Kothe C., Björk G., Inoue S. & Bourennane M. On the efficiency of quantum lithography. New J. Phys. 13, 043028 (2011).

[b29] Peeters W. H., Renema J. J. & van Exter M. P. Engineering of two-photon spatial quantum correlations behind a double slit. Phys. Rev. A 79, 043817 (2009).

[b30] Tsang M. Fundamental Quantum Limit to the Multiphoton Absorption Rate for Monochromatic Light. Phys. Rev. Lett. 101, 033602 (2008). 18764255 10.1103/PhysRevLett.101.033602

[b31] Leach J. *et al.* Quantum correlations in optical angle-orbital angular momentum variables. Science 329, 662 (2010). 20689014 10.1126/science.1190523

